# Delayed Presentation of Tetralogy of Fallot with Isolated Cyanosis

**DOI:** 10.1155/2018/7412869

**Published:** 2018-06-20

**Authors:** Zachary Zemore, Avni Sharma, Kerri Carter, Aline Baghdassarian

**Affiliations:** ^1^Departments of Emergency and Internal Medicine, VCU Health, Richmond, VA, USA; ^2^Department of Pediatrics, VCU Health, Richmond, VA, USA; ^3^Department of Pediatrics, Division of Cardiology, VCU Health, Richmond, VA, USA; ^4^Department of Emergency Medicine, Division of Pediatric Emergency Medicine, VCU Health, Richmond, VA, USA

## Abstract

A pediatric patient with hypoxia or cyanosis can frighten even the most seasoned emergency providers. Patients with these symptoms require immediate evaluation and intervention to stabilize their condition. While the differential can be broad, specific attention must be paid to cardiopulmonary etiologies. Tetralogy of Fallot is the most common cyanotic congenital heart abnormality, and routine screening surprisingly misses a significant amount of these cases. This case serves as an example of a missed diagnosis by screening efforts and reaffirms the resuscitation algorithm of a hypoxic pediatric patient that all emergency providers should be familiar with.

## 1. Introduction

Tetralogy of Fallot was first described as early as 1671 by Nils Stenson. However, it was not until 1888 that Etienne-Louis Fallot described the condition in detail, thus imprinting his namesake on the condition [1]. Tetralogy of Fallot was largely untreatable until 1944, which marked the initiation of modern surgical intervention for this condition. The operation performed by Alfred Blalock marked the first successful attempt to palliate Tetralogy of Fallot or any congenital cardiac disease [2]. Surgical intervention is now relatively commonplace and successful, allowing many children with this condition to live well into adulthood. Screening initiatives have since taken precedence, in an effort to identify and correct the underlying structural abnormalities prior to clinical decompensation. The following report describes a case of a child with a delayed presentation and isolated symptom of cyanosis, who was not identified by routine pre- and postnatal screening efforts.

## 2. Case Report

The 2-month-old African American infant was born at full term, at 2890 grams, following an uncomplicated pregnancy and delivery with an Apgar of seven and nine at one and five minutes, respectively. In the newborn nursery, her physical exam did not note any murmur, and her congenital cardiac screen was documented as normal (pre- and postductal saturations 100%) on day two of life. She had no family history of congenital heart defects, arrhythmias, or other cardiac diseases. The infant was discharged home with her mother on day of life two with follow-up arranged with the pediatrician.

At home, she remained stable with no respiratory distress, no feeding difficulties, and adequate weight gain. Her mother did note that the infant's hands and feet appeared slightly “dark” at home, but she was reassured that this was normal acrocyanosis. Her pediatrician saw her at two separate routine office visits, where no murmur or other abnormality was reported. Approximately three days prior to presentation, the infant developed cough, congestion, and rhinorrhea. She was due for her two-month well-child exam, so her mother took her to the clinic for an evaluation. At that time, the infant weighted 4.3 kg placing her at approximately the tenth percentile on a Center for Disease Control growth chart. The pediatrician was concerned by her visible acrocyanosis and pulse oximetry documented an oxygen saturation of 80% in room air, so she was immediately referred to an emergency department for further evaluation.

**Figure 1:**
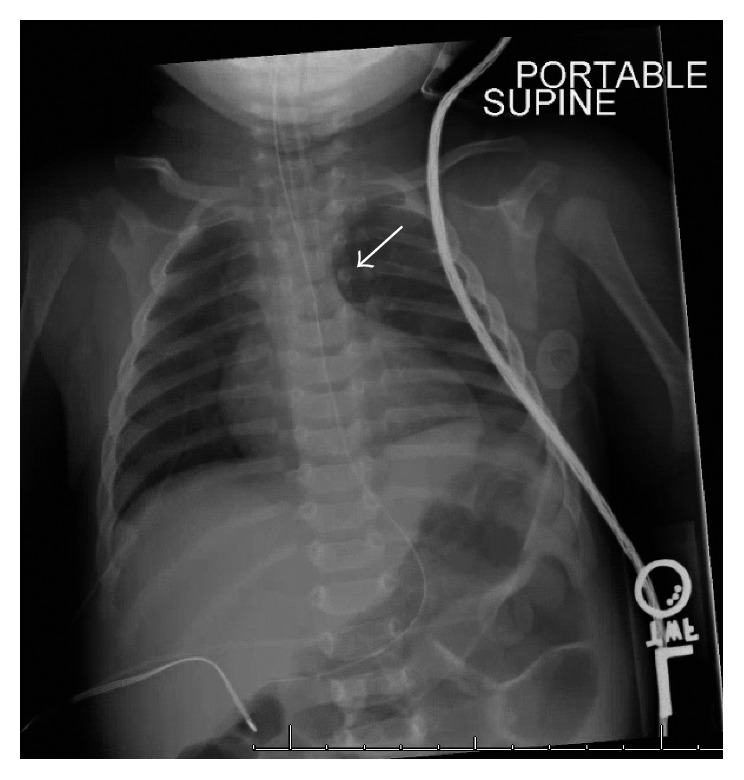
Portable X-ray which shows an absence of the aortic knob (arrow) and inability to visualize the descending aorta, suggestive of a right-sided aortic arch.

On arrival to the emergency department, she was hypoxic with oxygen saturations of 20–30% and hypothermic to 32°C. No murmurs were noted on physical exam. Peripheral access was established, first with a tibial intraosseous catheter and then with a peripheral intravenous line; she received a normal saline bolus, a blood culture was drawn (which remained negative), and a dose of ceftriaxone was administered. A rapid sequence intubation was performed with etomidate and vecuronium. The child was successfully intubated with no complications. Despite this and increasing oxygen administration up to 100% FiO_2_, her oxygen saturation peaked at 80%. Initial labs were significant for blood glucose 95 mg/dL, white blood cell count 7 × 10^9^/L, hemoglobin 22 g/dL, hematocrit 68%, and lactate 5.5 mmol/L. Chest X-ray could not view the aortic knob or descending aorta well and could not rule out a right-sided aortic arch (Figure 1). With these findings and the severity of her illness, the decision was made to transfer the patient to a tertiary pediatric medical center.

On arrival, a brief transthoracic echocardiogram (TTE) was concerning for Tetralogy of Fallot with critical pulmonic stenosis versus atresia. She received an additional normal saline bolus, sodium bicarbonate, midazolam, and fentanyl. On attempts to draw labs, her blood was noted to be very dark and hyperviscous, clotting almost immediately.

**Figure 2:**
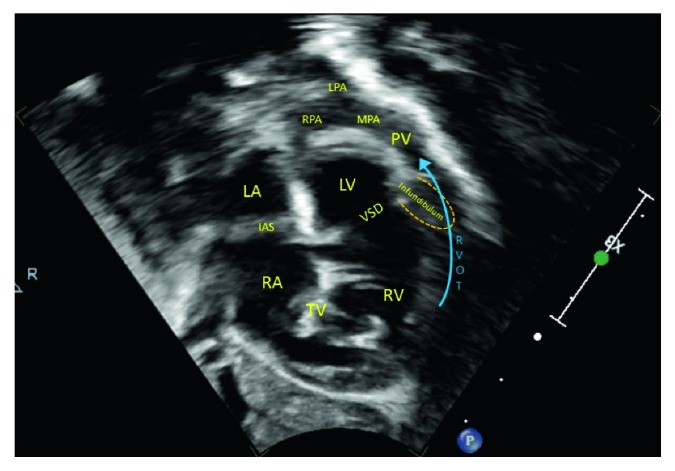
2D image in the subcostal right anterior oblique plane (“Tet view”) demonstrating the large VSD produced by anterior and superior malalignment of the infundibulum, which completely obstructs the RVOT producing profound cyanosis and the need for an emergent BT shunt. PV, MPA, and branches are hypoplastic, each measuring approximately 3 mm at the time of presentation. RA: right atrium, LA: left atrium, IAS: interatrial septum, TV: tricuspid valve, RV: right ventricle, LV: left ventricle, VSD: ventricular septal defect, RVOT: right ventricular outflow tract, PV: pulmonary valve, MPA: main pulmonary artery, RPA: right pulmonary artery, and LPA: left pulmonary artery.

The infant was admitted to the pediatric intensive care unit (PICU) for further stabilization and started on a prostaglandin drip with the hope of reopening her ductus arteriosus to increase pulmonary blood flow. Repeat TTE confirmed the diagnosis of Tetralogy of Fallot with severe pulmonary stenosis, hypoplastic main and branch pulmonary arteries, right-sided aortic arch, and small secundum atrial septal defect (Figure 2). At no point was a murmur heard by any provider from the ED, PICU, or Cardiology. Over the next several hours, she continued to decompensate with worsening hypoxia and hypotension despite phenylephrine infusion, fentanyl infusion, and aggressive fluid resuscitation. Pediatric cardiothoracic surgery evaluated the patient, and the decision was made to perform an emergent Blalock–Taussig (BT) shunt.

The patient underwent modified BT shunt with a 3.5 mm Gore-Tex shunt anastomosing the innominate artery to the main pulmonary artery. There were no complications, and she returned to the PICU for post-op recovery. Daily TTE demonstrated normal biventricular function without pericardial effusion, as well as flow across BT shunt and through both branch pulmonary arteries. On post-op day one, she was extubated and weaned to room air, where she was able to maintain oxygen saturations around 88%. By post-op day two, she was tolerating oral feeds. She was discharged home on post-op day four with 40.5 mg of aspirin daily to help prevent shunt thrombosis. On discharge, her parents noted a vast difference in the infant's general appearance and energy level. The plan is to take the infant back to the operating room to perform a complete repair with a transannular patch at 4–6 months of life.

## 3. Discussion

Tetralogy of Fallot is estimated to occur in as many as 1 in 3600 live births, making it the most common cyanotic congenital heart disease [3]. Classically, infants are diagnosed after birth but prior to discharge from the nursery, although in utero diagnosis is also possible now [4]. Infants are often noted to be cyanotic, have an audible heart murmur, and have decreased oxygen saturation on pulse oximetry testing. Screening for congenital heart disease is endorsed by the American Academy of Pediatrics among other professional societies and is even required by state law in many regions [5]. This is because congenital heart defects are the leading cause of infant mortality from birth defects, and early recognition and intervention can allow an affected infant to live well into adulthood [3, 6]. Even with screening, the diagnosis is delayed in over 10% of critical congenital heart disease cases [7]. Screening is aimed at diagnosing patients with critical congenital heart defects, thus smaller defects are likely missed at an increased frequency. Some patients with Tetralogy of Fallot initially do not have a large degree of right ventricular outlet obstruction, allowing them to oxygenate more effectively. When missed by screening, patients with Tetralogy of Fallot typically develop increasing hypoxia as their ductus arteriosus closes and they have progressive right ventricular outlet obstruction. A rapid reduction in blood flow across the right ventricular outlet could be one of several explanations for the isolated symptom of cyanosis observed in this case. If undiagnosed, the child can present critically ill and develop complications related to their condition ranging from respiratory distress to death.

Cyanosis can be more difficult to detect in dark-skinned individuals and is dependent on the absolute concentration of deoxygenated hemoglobin [7]. It can be useful to differentiate between central and peripheral cyanosis, also called acrocyanosis. Peripheral cyanosis is present in the distal extremities; it can be physiologic, appearing wherever there is a large arteriovenous oxygen gradient that develops with slow blood flow through distal capillary beds [8]. The etiology can be innocent, related to cold environmental temperatures, or other external factors. Any cause of cyanosis must be taken seriously, however, as peripheral cyanosis can also be caused by severe systemic illnesses such as sepsis, polycythemia, or hypoglycemia. Alternatively, central cyanosis involves the trunk of the body and classically presents on the mucous membranes or tongue. Central cyanosis is never physiologic and requires immediate evaluation, particularly for a cardiopulmonary etiology.

Upon presentation, a hypoxic child should immediately have vital signs taken, and a focused physical exam should be performed looking for the underlying etiology. Initial evaluation should focus on maintaining adequate circulation, airway, and breathing [9]. If the child has no pulse, cardiopulmonary resuscitation should be rapidly initiated. If there is concern for hypovolemia, intravenous access should be rapidly obtained, through an intraosseous catheter if necessary, which has been shown to be safe and effective [10]. These devices allow for rapid access and can be paramount when providing fluid resuscitation, medications, or obtaining labs. When pulses are present, the airway should be evaluated to determine the need for adjunctive airway devices such as supplemental oxygen or orotracheal intubation. Finally, breathing should be evaluated to determine if mechanical ventilation or other management such as needle decompression is required.

Due to the difficulty in determining the etiology of hypoxia and cyanosis, additional testing is frequently needed. Evaluation should focus on tests that help to elucidate one of the common causes of hypoxia and cyanosis (Table 1). Particular importance should be placed on obtaining blood sugar, complete blood count with differential, arterial blood gas, and calcium levels. Invaluable imaging includes chest X-ray and echocardiogram. Supplemental tests could include basic metabolic panel, electrocardiogram, blood cultures, and a hyperoxia test. In particular, the hyperoxia test can be of notable importance because it can help to determine between a cardiac and pulmonary etiology of hypoxemia. When a patient has oxygen saturation less than 85% in room air, this test is performed by administering 100% FiO_2_ for up to ten minutes and evaluating the change in oxygen saturation. It is performed on the right upper extremity to eliminate possible alterations from shunting [11]. With supplemental oxygen provided, a ventilation/perfusion mismatch would be improved, resulting in oxygen saturations greater than 97%, thus indicating likely pulmonary pathology. However, shunt physiology as is seen in many cardiac conditions would go relatively unchanged [12, 13]. Moreover, elevated hematocrit is a physiologic response to chronic hypoxia to increase the body's oxygen-carrying capacity and may suggest that the hypoxia is likely longstanding [14, 15].

This patient notably had a very elevated hematocrit and continued hypoxia despite 100% FiO_2_ administration, which is suggestive of a right-to-left shunt and a fixed obstruction to pulmonary blood flow as seen in Tetralogy of Fallot. Acute hypoxia in Tetralogy of Fallot is traditionally called a hypercyanotic spell or “Tet spell” and is precipitated by increased pulmonary vascular resistance (PVR) or decreased systemic vascular resistance (SVR) [16]. The patient was placed on a phenylephrine drip in an attempt to increase SVR, which would decrease right-to-left shunting and ultimately increase oxygenation. Fentanyl was chosen as the sedating agent to reduce PVR and limit effects on SVR compared to other agents.

When a congenital heart defect is diagnosed, consultation with cardiology and cardiothoracic surgery is paramount. In 25% of cases of Tetralogy of Fallot, there is a right-sided aortic arch [3], as was seen in this case. Additionally, there are numerous other conditions associated with Tetralogy of Fallot, such as anomalous origins of the coronary arteries or atrioventricular (AV) conduction tissues [16], cardiac arrhythmias [3], and multiple genetic abnormalities with potentially serious consequences. Patients with Tetralogy of Fallot are often screened for such defects with genetic testing [4]. If managed appropriately with surgical interventions and frequent follow-ups, patients have a high probability of survival. The 40-year survival rate is estimated to be upwards of 90% [3].

## 4. Conclusion

Delayed presentation of Tetralogy of Fallot occurs more often than realized and can be missed despite proper screening. Any infant presenting with hypoxia or cyanosis must be assessed for congenital heart disease among other conditions to evaluate for severe or reversible etiologies.

## Figures and Tables

**Table 1:** Differential diagnosis of hypoxia or cyanosis in an infant [7, 8, 13].

Congenital heart disease	Shock states	Hematologic causes	Neurologic causes
(i) Tetralogy of Fallot	(i) Cardiogenic	(i) Methemoglobinemia	(i) Seizures
(ii) Transposition of the great arteries	(ii) Hypovolemic	(ii) Anemia	(ii) Phrenic nerve palsy
(iii) Total anomalous pulmonary venous connection	(iii) Obstructive	(iii) Polycythema	
(iv) Tricuspid atresia	(iv) Distributive		
(v) Truncus arteriosus			

Acquired respiratory disease	Infectious respiratory disease	Congenital respiratory disease	Miscellaneous causes

(i) Trauma	(i) Laryngotracheitis	(i) Laryngomalacia	(i) Cold exposure
(ii) Foreign body	(ii) Epiglottitis	(ii) Choanal atresia	(ii) Breath-holding spells
(iii) Pneumothorax	(iii) Bacterial tracheitis	(iii) Micrognathia
(iv) Asthma	(iv) Pneumonia	(iv) Pulmonary hypoplasia
(v) Pulmonary edema	(v) Bronchiolitis	(v) Pierre Robin sequence
		(vi) Cystic fibrosis

## Data Availability

All data generated or analyzed during this study are included in this published article (and its supplementary information files).
